# MCM Paradox: Abundance of Eukaryotic Replicative Helicases and Genomic Integrity

**DOI:** 10.1155/2014/574850

**Published:** 2014-10-19

**Authors:** Mitali Das, Sunita Singh, Satyajit Pradhan, Gopeshwar Narayan

**Affiliations:** ^1^Cancer Genetics Laboratory, Department of Molecular and Human Genetics, Banaras Hindu University, Varanasi, India; ^2^Department of Zoology, Mahila Mahavidyalaya College, Banaras Hindu University, Varanasi, India; ^3^Department of Radiotherapy & Radiation Medicine, Banaras Hindu University, Varanasi, India

## Abstract

As a crucial component of DNA replication licensing system, minichromosome maintenance (MCM) 2–7 complex acts as the eukaryotic DNA replicative helicase. The six related MCM proteins form a heterohexamer and bind with ORC, CDC6, and Cdt1 to form the prereplication complex. Although the MCMs are well known as replicative helicases, their overabundance and distribution patterns on chromatin present a paradox called the “MCM paradox.” Several approaches had been taken to solve the MCM paradox and describe the purpose of excess MCMs distributed beyond the replication origins. Alternative functions of these MCMs rather than a helicase had also been proposed. This review focuses on several models and concepts generated to solve the MCM paradox coinciding with their helicase function and provides insight into the concept that excess MCMs are meant for licensing dormant origins as a backup during replication stress. Finally, we extend our view towards the effect of alteration of MCM level. Though an excess MCM constituent is needed for normal cells to withstand stress, there must be a delineation of the threshold level in normal and malignant cells. This review also outlooks the future prospects to better understand the MCM biology.

## 1. Introduction

In early 1980s, minichromosome maintenance (MCM) proteins were first identified in budding yeast* Saccharomyces cerevisiae *[1]. These mutant variants were defective in maintenance of minichromosome, a plasmid with a cloned centromere and replication origin [2, 3]. MCM proteins were thus found to have essential roles in DNA replication [4, 5]. Subsequent identification of proteins with a particular identical sequence termed “MCM box” [6] leads to the constitution of a protein family, the MCM family. The family consists of at least 6 homologues, MCM 2–7 [1], which is directly involved in eukaryotic DNA replication [7]. MCMs remain at the centre of interest of biochemists, geneticists, and cancer biologists since 30 years of their identification. The studies provide good knowledge of MCM structure, function, particular role in DNA replication, and abnormalities leading to cancer. On the other hand, some paradoxes came forward with the advancement of studies regarding the function of MCMs in DNA replication. It has been found that the MCM hexamers are loaded in excess onto chromatin and distributed on the locations distant from the origins. Solving the paradox known as the “MCM paradox” is challenging to the scientific community. In this review we have discussed the role of MCMs in DNA replication, concepts generated to solve the paradoxes, their extension to cancer biology, and future perspective.

## 2. MCMs as a Component of Replication Origins

MCM 2–7 is expressed in all eukaryotes [1]. Physical interaction between MCM 2–7 components was determined by several molecular techniques such as two-hybrid, coimmunoprecipitation and biochemical purification [8–11]. It has been proposed that MCM 2–7 makes a heterohexameric complex in 1 : 1 : 1 : 1 : 1 : 1 ratio. The core of MCM 2–7 complex comprising MCM 4, 6, 7 has DNA helicase activity [12–14]. The other three components of the complex may play other roles during replication such as MCM2 binds to histones and facilitate histone redeposition during DNA synthesis [15]. However, the fact that MCM proteins are essential in vertebrate DNA replication was confirmed by the studies in* Xenopus*, where MCM depleted egg extracts were unable to support further DNA synthesis [16, 17].

A parallel concept of “replication licensing” was emerging in 1980s from the observation that sperm chromatin in a nucleus replicated only once in a cell cycle and became competent again in mitosis but a specific DNA sequence is not required for replication initiation. This concept hypothesized that there must be a positive “license” to mark the replicated sequences from the unreplicated ones thus preventing the reinitiation of replication at the already replicated sites [18]. The cell cycle dependent change in subcellular localization of MCMs in yeast and* Xenopus* proposed these proteins to be replication licensing factors [8, 15, 19]. However, the replication licensing mechanism involves a series of events and a number of proteins. The first step of replication licensing mechanism is the formation of prereplication complex (pre-RC). Pre-RC formation involves assembly of a number of proteins such as origin recognition complex (ORC), Cdc6, Cdt1, and MCM 2–7. This in turn leads to a chain of events allowing binding of DNA polymerase and other several factors to chromatin to start DNA replication [7, 20–22].

## 3. MCMs Function as Helicases

After the identification of MCMs as replication licensing factors, their biochemical function at the chromatin had been searched for. The heterohexameric ring structure of MCM 2–7 suggests that this protein complex may work as replicative helicase. The entire MCM 2–7 complex in fission yeast or MCM (4, 6, 7)_2_ complex from the mouse shows a globular structure with a central channel of 3-4 nm similar to many multisubunit helicases from all kingdom of life. This channel can encircle either single or double stranded DNA [1]. In 2001, Labib and Diffley [23] enquired whether MCM 2–7 is the eukaryotic DNA replication fork helicase. The first evidence came from the study of Yukio Ishimi, where they had shown that purified MCM 4, 6, 7 complexes have 3′ → 5′ DNA unwinding capacity [24]. Consequently, they have also shown that mouse MCM 4, 6, 7 complexes have intrinsic DNA helicase activity [12]. Not only the MCM 4, 6, 7 subcomplex but a whole MCM 2–7 complex had been reported to have* in vitro* helicase activity where the MCM 2–5 junction acts as an ATP drive gap or “gate” [25]. Recent studies have shown that the MCM 2–7 proteins make a core of the replicative DNA helicase and that they are first loaded at replication origins in an inactive form. The helicase is then activated by recruitment of the Cdc45 and GINS proteins into a holo-helicase known as CMG (Cdc45, MCM 2–7, GINS) [26]. Identification of several interacting partners and the real action mechanism of this holo-helicase keeps researchers involved in studies of MCMs. Very recently it has been shown that the human CMG complex (hCMG) can unwind DNA duplex regions up to 500 bp hydrolyzing ATP and in combination with human DNA polymerase *ε* leads to the formation of oligonucleotide products up to >10 kb [27]. The same group has also identified human chromosome transmission fidelity 4 (hCTF4) as an interacting partner of hCMG complex [28].

The helicase activity of MCM proteins was also found in archeal species [29].* Methanobacterium thermoautotrohicum*, the bacterium, contains only one MCM protein which forms a homohexameric ring. The homohexamer contains a positively charged central channel of 2–4 nm to bind with DNA and act as a helicase [30–33]. A single MCM homolog from* Sulfolobus solfataricus* has been reported to form hexamers in solution and can dissolve 5′ tailed oligonucleotides by its helicase activity [34].

MCM 2–7 proteins are categorized in superfamily 6 (SF6) helicases [35, 36] in which each subunit of a hexameric helicase contains an AAA+ domain. In these helicases the ATPase sites are generated in between two adjacent subunits: one is providing the walker A and walker B motif for ATP binding and the other partner provides the arginine finger motif for ATP hydrolysis. MCM 2–7 is also related to viral homohexameric helicases (superfamily 3 (SF3)) including the SV40 large T antigen and papilloma virus E1 protein [26].

Boos et al. [26] also summarized the evidence, indicating MCM 2–7 as the engine of the replicative helicase. Firstly, MCM 2–7 travels with replication forks, forming part of the purified replisome progression complex (RPC) in yeast [36, 37] and vertebrates [38–41]. Secondly, acute inactivation of MCM 2–7 subunits during S phase results in rapid replisome inactivation [42, 43] and, third, purified yeast MCM 2–7 complex shows* in vitro* helicase activity [25].

## 4. The MCM Paradox

Above discussed lines of evidence substantially show that MCM 2–7 complex has helicase activity to unwind double stranded DNA and thus helps to initiate DNA replication. But there are some issues that prohibit accepting the role of MCM 2–7 as helicase. The issues termed as “MCM paradox” came from two observations: (a) eukaryotic MCM 2–7 complexes are not localized at the sites of replication like viral and bacterial helicases. MCM 2–7 complexes in higher eukaryotes are distributed over nonreplicated DNA rather than on replication forks; (b) the paradox is elaborated by another observation that excess MCM heterohexamers are loaded onto chromatin rather than replication origins and ORCs [44]. The basic questions were how MCM 2–7 complexes perform their function as helicase from a distance away from the replication sites and what the purpose of the excess bound MCMs onto chromatin more than the origins themselves is.

Madine et al. [45] have shown by immunofluorescence that* Xenopus* MCM3 does not colocalize with sites of DNA replication. The protein is almost uniformly distributed on chromatin and is suddenly lost during replication. Same line of evidence in human cells came from another study in 1996. The authors had used high resolution confocal microscopy to determine the subnuclear sites of chromatin-bound MCM proteins in comparison to the sites of replicating DNA. They found MCM proteins to be entirely nuclear in HeLa cells at interphase and to exist in nucleoplasmic and chromatin bound forms. The chromatin bound form is present throughout the nucleus in late G1 and early S and at discrete subnuclear sites following progression into S phase. Interestingly, as the S phase proceeds further, the MCMs are displaced from their site on replicating chromatin. Furthermore, the chromatin bound fraction was found to be absent in G2 and mitotic nuclei. The observation that MCMs are present on unreplicated chromatin rather than replicating one emphasized their role in monitoring the unreplicated chromatin to ensure only a single round of DNA replication [46]. In Chinese hamster ovary (CHO) fibroblasts, MCM2 associates with early and late replicating chromatin in late telophase. Subsequently, more MCM2 is associated to chromatin throughout the G1 phase and the maximum loading occurs at the G1/S transition. MCM2 was found to get dissociated from replicons shortly after their initiation [47]. In 2005, Mašata et al. [48] used a robust statistical method to be more accurate in analyzing the localization of MCM proteins and replication machineries. In HeLa cells, majority of MCM2 proteins do not colocalize with replication foci, but a very small but significant fraction of MCM2 signals colocalize with DNA replication foci throughout the S phase. The colocalization of MCM2 protein with replication foci during S phase thus explains an active function of MCM 2–7 complex in replication. Supporting the above findings, a recent* in vivo* licensing assay has proved that the concentration of chromatin bound MCM2 and MCM4 changes with progression of G1 phase and they fail to colocalize with other replication machinery [49].

Eukaryotic DNA replication starts at multiple sites throughout the genome. In budding yeast, the initiation sites, known as autonomously replicating sequences, are distributed all over the genome at an interval of every 40 kb. In somatic cells, replication can be initiated once every ~150 kb. In embryonic cells of* Xenopus laevis*, DNA replication initiation sites are regularly spaced at every 9–12 kb without sequence specificity [50]. In all cases the initiation is first marked by the binding of ORC [7]. As noted earlier, the second part of MCM paradox concerns the abundance of chromatin bound ORCs and MCM 2–7 complexes. In yeast,* Xenopus*, and highly proliferating human cancer cells HeLa, the number of MCM 2–7 complexes bound to chromatin highly exceeds the number of origins [10, 51–55]. In* Xenopus*, the number of MCM 2–7 complexes is found to be 10–40 times more than the actual initiation events are [56, 57]. More precisely, in* Xenopus*, the minimum length of DNA required to recruit ORC and MCM 2–7 is ~80 bp. The ratio of MCM 2–7 : ORC in this small fragment is perfectly 1 : 1. With a DNA fragment longer than 80 bp, the MCM 2–7 : ORC ratio increased up to 40 : 1 [58].

## 5. Proposed Models to Resolve “MCM Paradox” 

Several plausible mechanisms have been proposed to explain the function of MCM 2–7 as replication helicase from a distance away from the replication site. According to “MCM eviction” mechanism, there is always a minor MCM population that escapes immunodetection but is associated at replication forks and the rest major fraction is released from the replication forks upon replication initiation [47, 59], as a consequence of chromatin condensation [60]. A rotary pump model depicts that those MCM 2–7 complexes have always been loaded at a distance from an active replication fork and, like rotary motors, they translocate DNA along its axis by helical rotation causing unwinding at the constrained replication fork [61]. With little variation of rotary pump model, a ploughshare model has been proposed, where MCM helicase encircles and translocates on dsDNA. Unwinding of DNA double strand occurred through a rigid “ploughshare” present at the trailing edge of the helicase that is inserted in between DNA double strands and cleaves the duplex. The model also assumes that multiple pairs of MCM 2–7 hexamers bind to each replicon in G1 phase but only a single pair is activated to perform as helicase [62]. All the models have a fact in common that there must be at least a few active MCMs at the replication fork. The fraction of these MCMs at replication forks may be undetectable in comparison to the detectable fraction present on chromatin distant from the active replication sites. Together with the ideas and observations, the current model of MCM action has been proposed by Aparicio et al. [59]. The group found that, in HeLa cell nuclei, the MCM proteins, at G1 phase, first concentrate on chromatin structures that subsequently become replication forks at S phase. The concentration of bound MCMs at replication forks gets reduced after replication initiation leaving a very small but detectable fraction still bound to the forks during the S phase, supposed to be the helicase. The findings were similar to those of a previous study [63]. The authors proposed an extension of “MCM eviction” mechanism of MCM dynamics based on the “loop model” of mammalian replication factories [59, 64]. According to this model, at a very early onset of G1, the MCM 2–7 complexes start to be loaded onto origin DNA in an ORC-Cdc6-Cdt1-dependent manner and spread all over the adjacent sequences. The amount of MCMs to be loaded on each origin may differ influencing their firing ability. At G1-S boundary, only a few MCM 2–7 complexes get activated by Cdc45, GINS, and other protein factors. These activated MCMs become able to escape the posttranslational modification signals that can cause their dissociation from chromatin and remain bound to both active and dormant origins upon initiation of DNA synthesis. In a multireplicon chromatin arrangement, several replicon units remain organized in cohesion-stabilized loops to form a rosette structure. Most of the origins are distributed at the base of the loops confining replication event at the central part of the rosette. The activation of replication forks leads to the MCM-BP mediated dissociation of inactive MCMs [65] from chromatin and continuous entry of PCNA. A small fraction of MCMs remain always bound to the active site of the DNA replication. Thus, the first part of the MCM paradox has been solved [59].

## 6. Excess MCMs Are Meant to Survive Replicative Stress

The second part of the MCM paradox concerns the purpose of quantitative excess of MCMs over origins. The common approach was to reduce the dosage of MCMs in replicating cells and analyze the posteffects. In yeast, it has been found that depletion of MCM2 does not affect their growth rate but a 50% reduction of the dosage of MCM2 in yeast decreases the number of origins to be used per replication event. There was an indication that the excess MCM proteins may be required for successful initiation at all replication origins [10]. Instead of hampering cell cycle, depletion of MCM7 causes activation of checkpoint signalling in human cancer cells, suggesting their roles in replication checkpoint signalling or activation of replication origins at the time of incomplete DNA replication [66, 67]. It is evident that MCMs may have an alternative role related to DNA replication; however, it remained difficult to conclude due to the lack of missing link(s). The concept of “dormant” origins has been proposed to be the missing link based on the origins that remain suppressed during normal DNA replication and can get activated and support initiation when replication forks are stalled in response to replicative stresses [68, 69]. In* Xenopus laevis*, there are 10 dormant origins distributed adjacent to each active origin. Both the active and dormant origins are bound by MCM 2–7 complexes and have potential to carry out replication process. But the dormant origins remain inactivated during a normal replication event by ATR mediated signalling pathway. In a stressed replication condition, such as in presence of aphidicolin, when the ATR signalling is inhibited by caffeine, an ATR-kinase inhibitor, the dormant origins get activated and partially rescue the replication process. Consequently, RNAi-based partial depletion of MCMs in* Caenorhabditis elegans* does not show any observable effect on cell viability or proliferation but becomes lethally hypersensitive to a nontoxic dose of replication inhibitor hydroxyurea. These observations suggested the role of excess MCM 2–7 complex in licensing dormant origins during replication stress [68]. Extension of similar studies in human cancer cells shows that RNAi mediated MCM5 silencing allows the cells to grow at a normal rate but makes these MCM depleted cells vulnerable to any kind of replication stress such as hydroxyurea or aphidicolin. The authors suggest that excess MCM 2–7 provides the cells safeguards against replicative stress by licensing dormant origins [70]. An extensive study using siRNA against each component of MCM 2–7 complex in human cancer cell lines was concordant with the above findings. Acute downregulation of MCM2 and MCM3 permits the formation of a limited but sufficient amount of MCM 2–7 complex, allowing the cells to proliferate. But their hypersensitivity to replication stress shows increased chromosomal aberrations and eventually raises a checkpoint response to block mitotic division. The acute downregulation of MCM 4–7, on the other hand, may reduce the number of chromatin bound MCM 2–7 complexes beyond the threshold and thus becomes fatal. It has been suggested that the excess MCM 2–7 complexes license dormant origins as a backup mechanism to maintain genomic integrity during replication stress [71]. The consequences of attenuated MCM expression in whole animal were studied using mouse model. Chaos3 (chromosomal aberrations occurring spontaneously 3) is a murine chromosomal mutation of MCM4. The viable mutant allele MCM4^Chaos3^ produces the polypeptide with a change from wild type phenylalanine to isoleucine at residue 345 (F345I), producing a destabilized form of MCM4. MCM4^Chaos3/Chaos3^ female mice develop mammary adenocarcinoma after 12-month latency. Hence, mutant MCM4 causes cancer predisposition. The embryonic fibroblasts (MEFs) from these mutant mice showed increased rate of DNA breaks after treatment of aphidicolin [72]. MEFs, containing hypomorphic MCM4^Chaos3/Chaos3^, show approximately 40% reduction of all MCM 2–7 expression. Depletion of MCM2, -6, or -7 in these mutant cells resulted in deleterious effects such as growth retardation, inhibited cell proliferation, genomic instability, and cancer predisposition. However, heterozygous condition of MCM4^Chaos3^ with normal MCM3 rescued the normal phenotype by increased chromatin bound MCMs [73]. MEFs from MCM2^IRES-CreERT2/IRES-CreERT2^ mice show decreased origin use in presence of hydroxyurea and are thus hypersensitive to these replicative stresses [74]. Both of the studies indicate that* in vivo* a cellular high level of MCMs is required for licensing active origins and maintenance of dormant origins as a backup in distress. However, excess MCMs are not only meant to license dormant origins but also take part in recombinational repair under replication stress as reported in fission yeast [75].

**Figure 1:**
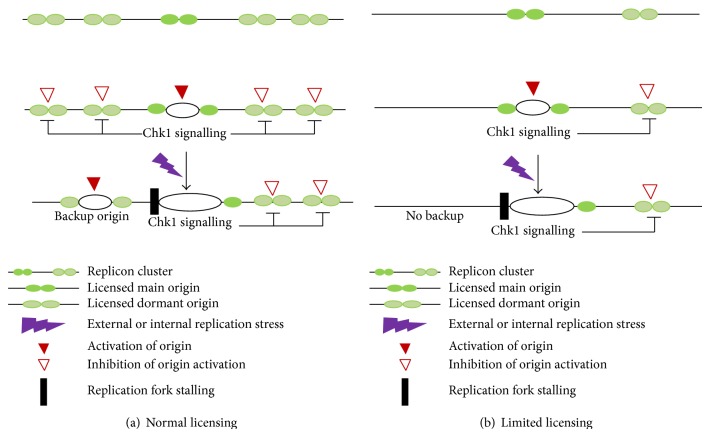
Purpose of excess MCMs: to license dormant origins as a backup mechanism for stress: (a) prior to S phase, the MCMs license main origins as well as many dormant origins which remain prevented to be fired by Chk1 signalling. At the time of stress, when replication forks get stalled, the backup origins get activated and continue the replication process; (b) in case of a limited licensing condition, at the time of stress there is no backup to retrieve the replication process; hence, the cells cannot withstand the stress (adapted from [71]).

It is evident that the replication licensing mechanism ensures the nonlicensing of a single new origin at the onset of S phase to prevent rereplication [76, 77]. In a subsequent S phase, if the cells face accidental problems such as replication fork stalling or disassembly, they cannot be retrieved. Cells therefore keep backup licensed dormant origins which are not used normally but are used when replication is at stake [69, 78]. So the most plausible explanation for the “MCM paradox” is that cells maintain abundant MCM 2–7 complexes distributed over the chromatin as a backup licensing mechanism to combat natural replicative stress generated during S phase (Figure 1).

## 7. Effects of MCM Attenuation* In Vivo*

The depleted level of MCMs has been proved to be deleterious for vertebrate systems. Positional cloning of mutated MCM5 in embryonic cells of Zebra fish leads to impaired retinal development characterized by deregulated cell cycle and increased rate of apoptosis. The mutation was applied in the differentiated cells at the third day of development, but the effect was restricted to those retinal stem cells hampering development of retina, tectum, and hindbrain. This indicates that different tissues respond differentially to MCM5 depletion [79]. Mutation in MCM4 was reported for the first time in human population in 2012. A frameshift mutation leads to the formation of truncated MCM4 protein. The mutation occurs due to the insertion of a single nucleotide prior to exon 2 thus shifting the splice acceptor site. This mutation is associated with a diseased phenotype called familial glucocorticoid deficiency characterized with poor adrenal development, short stature, and NK cell deficiency and increased DNA breakage [80, 81]. However, there is no clear report of cancer predisposition in these patients. The consequences of MCM2 and MCM4 mutations were discussed in previous section [73, 74]. There is another example of dominant mutant allele Mcm4^D573H^ which  has been found to be associated with spontaneous dominant leukemia in mice. These leukemic mice have a normal level of MCM proteins but MCM4^D573H^ is unable to incorporate into MCM 2–7 complexes causing chromosomal abnormalities leading to cancer [82]. Mouse embryos homozygous for a mutant MCM2 (Mcm2^IRES-CreERT2/IRES-CreERT2^) allele develop normally but can survive only for 10–12 weeks. They acquire severe impairment of tissue development due to stem cell deficiency, accumulate huge chromosomal abnormalities, and immediately die of cancer [83]. MCM2 depletion causes a high rate of thymic lymphoblastic lymphoma characterized with manifold genomic instabilities. The most common of these chromosomal abnormalities is a deletion of a genomic region of approximately 0.5 Mb altering expression of several genes contributing to the development of cancer [84]. Above discussed citations exemplify the biochemical, cellular, and phenotypic consequences of limited licensing in the form of impeded MCM loading increased genomic instability and an ultimate formation of cancer, respectively [69].

## 8. MCM Expression and Cancer

MCMs are found to be overexpressed in multiple cancers such as in prostate cancer [85], colon cancer [86], meningioma [87], breast carcinoma [88], medulloblastoma [89], gastric adenocarcinoma [90], cervical cancer [91, 92], osteosarcoma [93], and esophageal squamous cell carcinoma [94]. Aberrant expression of individual components of MCM 2–7 complex makes them suitable biomarkers for malignancies [95]. MCM2 is a proposed biomarker in oligodendrioma [96], renal cell carcinoma [97], esophageal squamous cell carcinoma [98], laryngeal carcinoma [99], breast cancer [100, 101], large B cell lymphoma [102], oral cancer [103], ovarian cancer [104], and gastric cancer [105]. MCM3 is a proliferative marker in papillary thyroid carcinoma [106]. MCM4 is a proliferation marker of nonsmall cell lung cancer [107]. MCM5 is a prognostic marker of ovarian cancer and prostate cancer [104, 108]. MCM6 protein level in plasma of hepatocellular carcinoma patients may be a novel biomarker for the malignancy [109]. Deregulated expression of MCM7 is a potential biomarker for colorectal cancer [110], small lung adenocarcinoma [111], and oral squamous cell carcinoma [112].

Hence, the overexpression of MCM genes can define the proliferative stage of a cell [113]. The reason behind deregulated expression of these proteins is still an open question. There are two possible explanations. One is that, due to an inappropriate action of CDKs, the malignant cells lost the power to differentiate, hence continuously divide and express MCMs. MCMs, in this case, are not directly oncogenic but mark the proliferative status of the cells. Another possibility is that deregulation of replication licensing system itself causes the development of cancer by inducing relicensing of replicated DNA promoting chromosomal instability [114].

Aneuploidy is an obvious consequence of inappropriate replication licensing. The overexpression of MCM2 along with other licensing proteins is significantly associated with aneuploidy to cause penile cancer [115]. The cervical cancer cell lines harboring high level of aneuploidy show overexpression of MCMs [92, 116]. Dosage alterations of MCMs have vivid cytogenetic background. The 3q and 8q containing MCM2 and MCM4, respectively, have been reported to show genomic amplification and chromosomal gain [117, 118]. MCMs, thus, may cytogenetically control their expression by a positive feedback loop participating in the “vicious cycle” [119] of aneuploidy and chromosomal instability.

Inhibition of MCMs on cancer cells has also been proved to be deleterious. A geminin mediated reduction in the chromatin bound form of MCM2 promotes cell type specific checkpoint response. A p53+/Rb+ cell, U2OS, showed an early S phase arrest by activation of intra-S phase checkpoints. On the other hand, p53−/Rb− cell line, Saos2, showed an accumulation in late S and G2-M phase and loss of intra-S phase checkpoints. In both cases the ultimate consequence was apoptosis. In IMR90 primary fibroblasts, overexpression of geminin caused G1 arrest and did not lead to apoptosis. A “licensing checkpoint” thus may take care of the primary cells with insufficient licensed origins and prevent them from proceeding into S phase [120]. Similar results were reported where inhibition of MCM2 by RNAi produces apoptotic response in P53+ and P53− cancer cell lines but causes G1 arrest in normal cells [121]. Trichostatin A and siRNA mediated downregulation of MCM2 generated apoptotic response in HCT116 cells [122]. Knocking down MCM7 not only inhibits cell proliferation in glioblastoma multiforme tumor cells but also prevents tumor growth in mouse models of glioblastoma multiforme [123].

## 9. Conclusion and Perspective

**Figure 2:**
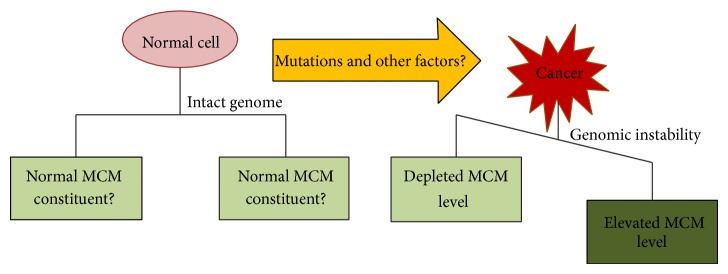
The unavoidable consequence of change in MCM level is genomic instability which may result in cancer. The threshold level of MCMs for genome maintenance of normal cells is still to be quantified and the factors causing aberrations in MCM level are yet to be identified.

It is evident from the above discussion that either elevation or depletion of MCM level causes genomic instability and thus consequently cancer (Figure 2). From the concept of MCM paradox and its solution we come to a conclusion that a high level of MCMs is needed by a cell normally to maintain the genomic constitution. There is a gap of knowledge as the proper quantification of the threshold level of MCMs, below or above of which is fatal, remains to be filled. Since cancer cells with elevated level of MCMs grow faster, there must be a difference in usage of dormant and main licensed origins; enlightenment in this area will help understand the replication mechanism of cancer cells better. As noted earlier that the licensing checkpoints respond differently in cancer and normal cells [119], more information is required for the complete designing of licensing checkpoint signalling. MCMs are placed downstream to many signal transduction pathways [124, 125] and are also reported to be controlled by miRNA [126, 127]. It will be very interesting to record the crosstalk between several signal transduction pathways controlling MCM expression and map a complex signalling network of DNA replication licensing.

Besides DNA replication licensing, MCMs are also actively involved in transcription process. MCM 2–7 complex components are also identified as the components of RNA polymerase II transcriptional apparatus [128]. It has been demonstrated that MCM5 is indispensable as a signal transducer and activator of transcription 1 (Stat 1) mediated transcriptional activation [129]. Recently, Hubbi et al. presented another line of evidence suggesting that the abundance of MCM proteins must be meant for functions other than DNA helicase activity. They demonstrate that MCM 2, 3, 5 and MCM 7 proteins directly interact with hypoxia-inducible factor-1 (HIF-1) *α* subunit to inhibit HIF-1 transcriptional activity by oxygen dependent manner. Thus, MCM proteins play important role in regulating transcription during oxidative stress [130]. Furthermore, the existence of filamentous structure made of excess MCMs located distally from origins with a capacity of chromatin remodeling [131] extends MCM paradox towards the solution with alternative functions of MCMs in DNA metabolism.
